# Repurposing population genetics data to discern genomic architecture: A case study of linkage cohort detection in mountain pine beetle (*Dendroctonus ponderosae*)

**DOI:** 10.1002/ece3.4803

**Published:** 2018-12-26

**Authors:** Stephen A. L. Trevoy, Jasmine K. Janes, Kevin Muirhead, Felix A. H. Sperling

**Affiliations:** ^1^ Department of Biological Sciences University of Alberta Edmonton Alberta Canada; ^2^ School of Environmental & Rural Sciences University of New England Armidale New South Wales Australia; ^3^ Biology Department Vancouver Island University Nanaimo British Columbia Canada

**Keywords:** genomic architecture, linkage disequilibrium, population genomics

## Abstract

Genetic surveys of the population structure of species can be used as resources for exploring their genomic architecture. By adjusting filtering assumptions, genome‐wide single‐nucleotide polymorphism (SNP) datasets can be reused to give new insights into the genetic basis of divergence and speciation without targeted resampling of specimens. Filtering only for missing data and minor allele frequency, we used a combination of principal components analysis and linkage disequilibrium network analysis to distinguish three cohorts of variable SNPs in the mountain pine beetle in western Canada, including one that was sex‐linked and one that was geographically associated. These marker cohorts indicate genomically localized differentiation, and their detection demonstrates an accessible and intuitive method for discovering potential islands of genomic divergence without a priori knowledge of a species’ genomic architecture. Thus, this method has utility for directly addressing the genomic architecture of species and generating new hypotheses for functional research.

## 1 INTRODUCTION

Advances in high throughput sequencing have made cost‐effective genotyping of thousands of single‐nucleotide polymorphisms (SNPs) possible, allowing a proliferation of population genetics studies (e.g., Baird et al., 2008; Davey et al., 2011; Elshire et al., 2011). Typically, these data are filtered to remove spurious signals, caused by sequence error or repetitive signal, to provide a consistent approach for assessing population genetic structure and a means of comparing datasets (Nielsen, Paul, Albrechtsen, & Song, 2011; Slate et al., 2009). However, population genetics studies are concerned primarily with assessing differences between independent markers, often neglecting potential insight into gene function and genomic architecture that can be found in co‐related loci (Luikart, England, Tallmon, Jordan, & Taberlet, 2003; Stinchcombe & Hoekstra, 2008).

A typical study of population structure with SNP data entails the use of three widely applied filtering procedures: (a) minor allele frequency (MAF) cutoffs to reduce the impact of rare alleles or genotyping errors in a population‐level analysis (Bagley, Sousa, Niemiller, & Linnen, 2017; Malenfant, Coltman, & Davis, 2015); (b) conformance to Hardy–Weinberg equilibrium (HWE) proportions to detect potential genotyping errors and support the assumption of neutrality in most markers (Hosking et al., 2004); and (c) linkage disequilibrium (LD) filtering to ensure independence of loci and remove repetitive genetic signal (Baird, 2015; Barton, 2011; Lu et al., 2016; Schilling et al., 2014). These methods are not consistently applied, however, and filtering is evaluated on a case‐by‐case basis depending on research needs and study species (Arnold, Corbet‐Detig, Hartl, & Bomblies, 2013; Narum, Buerkle, Davey, Miller, & Hohenlohe, 2013). Although neutral markers are useful for investigations of genetic drift and gene flow, recent work has called into question the value of removing non‐neutral markers in SNP assays (Batista, Janes, Boone, Murray, & Sperling, 2016; Helyar et al., 2011). Likewise, filtering out repetitive markers in SNP datasets may prevent useful genetic signal from being overwhelmed by a few linked markers, but can hinder the reconciliation of genetic differentiation with genomic architecture.

Islands of genomic differentiation, or “speciation islands,” are defined as areas within a genome that have higher allelic variance between populations, most commonly measured by *F*_ST_ (Turner, Hahn, & Nuzhdin, 2005; Wolf & Ellegren, 2017). The validity of islands of genomic differentiation is a topic of ongoing debate (Hahn, White, Muir, & Besansky, 2012; Michel et al., 2010; Noor & Bennett, 2009). Researchers have observed that markers diverge between populations at different rates in localized genomic regions, but the role that heterogenous genomic regions play in speciation—whether causative, symptomatic, or unrelated—is unclear. Nevertheless, genomic islands of differentiation have become an attractive concept to explain how species boundaries are formed and maintained between sympatric and parapatric populations (Marques et al., 2016; Wolf & Ellegren, 2017).

The traditional approach for detecting islands of genomic differentiation, known as genome scanning, uses a sliding window of *F*_ST_ calculations along the length of a genome. However, application of this method is restricted to organisms for which large, contiguous genome sequences have been assembled and is of limited use for the many species with minimal genomic resources (Feulner et al., 2015; Renaut et al., 2013; Turner et al., 2005). Kemppainen et al. (2015) recently released a tool for calculating linkage disequilibrium (LDna) that uses network analytical tools to visualize groups of linked loci across a genome. LDna has been used to reduce data dimensionality while searching for QTLs in model organisms (Li, Kemppainen, Rastas, & Merila, 2018) and can provide evidence of inversions and islands of genomic differentiation (Benestan et al., 2016; Lindtke et al., 2017; Ravinet et al., 2017). In this paper, we employ a similar approach to reduce dimensionality in our data while looking for cohorts of linked markers undergoing divergence or directional selection.

**Figure 1:**
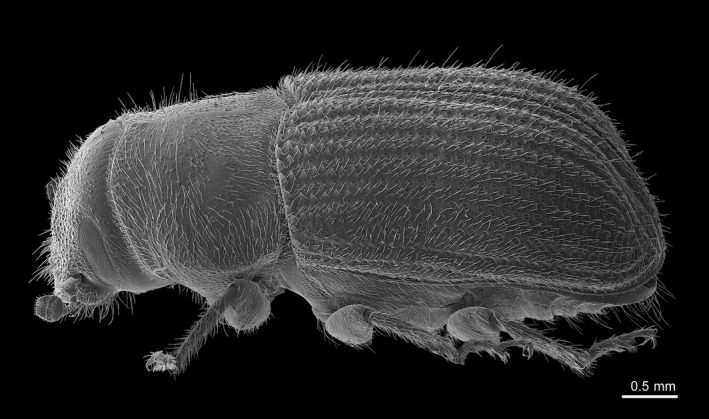
The mountain pine beetle (*Dendroctonus ponderosae*). Scanning electron micrograph was taken by Jack Scott and is used with permission of the TRIA project

One species of interest for speciation processes is the mountain pine beetle (MPB, *Dendroctonus ponderosae* Hopkins: Curculionidae, Scolytinae; Figure 1), an irruptive forest pest that has devastated millions of hectares of productive forest within western Canada and the United States (Bentz et al., 2010; Safranyik & Carroll, 2006; Safranyik et al., 2010). Evidence of incipient speciation has been found in US populations surrounding the Great Basin, where three distinct Y‐haplotypes result in hybrid male sterility in experimental crosses (Bracewell, Bentz, Sullivan, & Good, 2017; Dowle et al., 2017). These speciation events are driven by rapid degradation of the neo‐Y chromosome proceeding independently between populations. In addition to rapid changes in sex chromosomes, changes in climate have expanded MPB's Canadian range northward and eastward into naive landscapes and host plants, providing an opportunity for adaptive radiation (Carroll, Taylor, Regniere, & Safranyik, 2003; Cullingham, Roe, Sperling, & Coltman, 2012; Fauria & Johnson, 2009; Janes et al., 2014). Within the beetle's Canadian range, MPB population genetic structure has a well‐defined north–south division (Batista et al., 2016; Cullingham et al., 2012; Janes et al., 2014; Mock et al., 2007; Samarasekera et al., 2012), but lacks fine‐scale population structure (Janes et al., 2016).

In addition to markers that have allowed extensive population genetics research, modest genomic resources exist for the investigation of MPB genomic architecture. Draft genomes for both a male and female MPB are available, but the sequences are distributed across 8,188 and 6,520 scaffolds, respectively (Keeling et al., 2013), and only a few gene families have been annotated (Fraser, Bonnett, Keeling, & Huber, 2017). Research into MPB gene function is aided by comparisons with resources for related species (McKenna et al., 2016; Richards et al., 2008; Vega et al., 2015), and the MPB genome has considerable synteny with that of the red flour beetle (*Tribolium castaneum *Herbst) (Keeling et al., 2013). Synteny has been historically defined as any two genes located on a single chromosome, but has now shifted to mean orthologous genes located in the genomes of separate species and sharing common descent (Passarge, Horsthemke, & Farber, 1999). For the purposes of this paper, we use the most recent sense of the term.

The MPB genome is characterized by a karyotype of 11AA + neo‐XY (Lanier & Wood, 1968). Neo‐XY sex‐determination arises when an X chromosome fuses with an autosomal chromatid, accompanied by the subsequent loss of the original Y chromosome (Bracewell et al., 2017; Kaiser & Bachtrog, 2010). The remaining unfused autosomal chromatid then functions as the neo‐Y chromosome, becoming a paralogue to part of the neo‐X chromosome. Autosomal fusion with sex chromosomes is relatively common in nature (Graves, 1998; Henzel et al., 2011; Watson, Spencer, Riggs, & Graves, 1991), and five of the seventeen karyotyped species within *Dendroctonus* possess a neo‐XY mechanism (Lanier, 1981; Zúñiga, Cisneros, Hayes, & Macias‐Samano, 2002). However, the 11AA+neo‐XY karyotype, in which the neo‐XY is derived from fusion with ancestral autosome 1, is unique to *D. ponderosae* and its sister species, *Dendroctonus jeffreyi* (Jeffrey pine beetle; Hopkins) (Reeve, Anderson, & Kelley, 2012; Víctor & Zúñiga, 2015).

Our study examines the genomic architecture of MPB using a genome‐wide set of SNPs originally developed to survey population structure (Trevoy, Janes, & Sperling, 2018). Previous exploration of sex chromosome evolution in MPB has provided insight into species delimitation, evolutionary biology, and population dynamics (Bracewell et al., 2017; Dowle et al., 2017). We employ an approach to data filtering that uses multivariate analyses to find additional cohorts of linked SNP markers in the MPB genome, highlighting potential islands of genomic differentiation.

## 2 METHODS

### 2.1 Sampling

A total of 205 wild MPB specimens were selected from 39 sampling events across British Columbia, Alberta, and the northwest USA between 2005 and 2015. Larvae (*N* = 139) and adults (*N* = 66) were field collected and either placed in 95% ethanol before being stored at −20°C or immediately stored at −80°C. Wild‐collected specimens were not sexed prior to DNA extraction. An additional 13 adults from north–south controlled crosses were captive‐reared. Further details concerning wild and laboratory‐bred specimens are given in Trevoy et al. (2018). To aid in the molecular identification of sex‐related markers, the 13 offspring from laboratory crosses were morphologically sexed by inspection of the sclerotized plectrum found on the beetle's seventh abdominal tergite (Lyon, 1958; Rosenberger, Venette, & Aukema, 2016; Safranyik & Carroll, 2006).

### 2.2 Library preparation

DNA extraction and library preparation methods followed Campbell, Davis, Dupuis, Muirhead, and Sperling (2017). Extractions from the 2005–2014 samples (Run 1) were sent to l'Institut de Biologie Intégrative et des Systems (IBIS) at Laval University for library preparation and sequencing on an Illumina HiSeq 2000 platform to produce 100 bp single‐end sequences. The 2015 and laboratory‐bred samples (Run 2) were extracted and sequenced at the University of Alberta Molecular Biology Services Unit (MBSU) in Edmonton, Alberta, on an Illumina NextSeq500 platform to produce 75 bp single‐end sequences. DNA extraction was identical for both runs, but library preparation differed; Run 2 was completed without data normalization or complexity reduction steps.

### 2.3 Data assembly and alignment

FastQC v0.11.05 (Andrews, 2010) was used to view the Illumina sequences and to ensure quality. Reads were demultiplexed using the STACKS v1.41 GBS pipeline (Catchen, Hohenlohe, Bassham, Amores, & Cresko, 2013) and custom wrapper scripts written in PERL (see Data Accessibility). We trimmed index‐sequence and *PstI* barcode sequence using Cutadapt v1.10 (Martin, 2011) to produce reads at a uniform insert size of 62 bp for both GBS runs, as STACKS requires uniform length for variant detection (Catchen et al., 2013). Individuals were aligned separately to both the female and male MPB draft genomes (Keeling et al., 2013) using BWA‐MEM v0.7.12 (Li & Durbin, 2009). Reads that did not map uniquely to the draft genome were discarded (BWA‐MEM option −c = 1), but split hits with fewer than four unique mapping regions were marked as secondary. These secondary hits, along with any chimeric reads, were removed with SAMtools v1.3 (Li et al., 2009). Both male‐ and female‐aligned data assemblies were run through the STACKS v1.41 refgen pipeline in order to generate the male and female SNP libraries. Default settings were used, except for a minimum read depth of 7.

### 2.4 Data filtering

First, to retain a reliable dataset for further analysis, we removed low‐quality individuals using VCFtools v0.1.12b (Danecek et al., 2011). Individuals were deemed unsuitable if they were missing data at >20% of genotyped loci when filtering loci for 20% maximum missing data (MM). Second, we performed additional filtering of the male‐ and female‐aligned datasets to remove loci with >5% MM and <5% MAF using only the female draft genome as a reference. We chose to focus on the female genome because it contains 20% fewer scaffolds but is 3.5% larger than the male draft genome, making it the less fragmented of the two draft genomes (Keeling et al., 2013). Third, LDHeatmap v 0.99‐2 (Shin, Blay, McNeney, & Graham, 2006) was used to filter the male‐ and female‐aligned datasets for HWE proportions and LD associations. A Bonferroni correction was applied to HWE (*p* = 2.5 × 10^−5^), while LD filtering used a cutoff of *r* ^2 ^= 0.5. LDHeatmap was chosen because it can calculate LD without known positions for markers; thus, it can detect LD even among high numbers of potentially unlinked scaffolds. The default assumption of 1 kbp separation between markers was used as per the LD Heatmap manual.

In this way, three filtered datasets were obtained for each of the male‐ and female‐aligned datasets: (a) filtered for high‐quality samples only (referred to as unfiltered); (b) the filtered dataset with 5% MM and 5% MAF filtering applied to loci (referred to as 5%‐only); and (c) the 5% filtered dataset with both HWE and LD filtering applied (referred to as FF, fully filtered) (Table 1). For subsequent analyses, we use the 5%‐only and FF datasets.

**Table 1:** Locus counts for the SNP dataset of 175 wild‐caught and 13 laboratory‐bred MPB after various filtering treatments. Cutoffs were set to 5% for maximum missing (MM) data, 5% for minor allele frequency (MAF), *p* = 0.000025 for Hardy–Weinberg equilibrium (HWE), and *r* ^2 ^= 0.5 for linkage disequilibrium (LD). Final analysis refers to analysis after filtering

Treatment	MM	MAF	HWE	LD	Female	Male	Final analysis	Results
Unfiltered	0	0	0	0	18,503	18,499	—	—
5%‐only	1	1	0	0	2,077	1,908	PCA, LDna	Figures 2b, 1c,d Figures 2, 3, 4
FF	1	1	1	1	1,480	1,488	PCA	Figure 2a

### 2.5 Multivariate analyses

Principal component analysis (PCA) is a widely used multivariate technique for compressing and distilling complex observations into intercorrelated orthogonal variables, called principal components (PC) (Abdi & Williams, 2010). Using ade4 (Dray, Dufour & Chessel 2007) in R (R Development Core Team, 2008), we performed a PCA on both the 5%‐only and FF datasets. The 13 laboratory‐bred individuals were grafted onto the analysis after calculating the PCs, so that laboratory‐bred specimens would not influence overall results. To identify SNP cohorts of potential functional or structural interest within the 5%‐only dataset, we plotted SNPs in descending order of PC loading values for the first four axes. Plateaus or steep declines in PC loading were used to delimit groups of SNPs with strong and uniform influence on each PC axis. The scaffold locations and clustering behavior of these cohorts were then assessed.

### 2.6 LDna

The 5%‐only dataset was used in LDna (Kemppainen et al., 2015) to explore cohorts of high LD within the dataset, as a means of visualizing results from LD Heatmap and further scrutinizing patterns of LD in our data. LDna presents loci as vertices, and LD as edges between vertices, to graphically represent linkage between genetic markers along increasing levels of LD stringency, calculated using *r* ^2^. LD network analyses used default settings (minimum of 10 edges to define cohorts; phi (*Φ*) = 2). LDna was not applied to the FF dataset since it had already been filtered for LD using LD Heatmap. The SNP compositions of the cohorts from LDna analyses were then compared to the SNP groups that were identified by high PC loading values.

### 2.7 BLAST+ and BLAST2GO

In order to identify the SNPs that influence PCs 1–4, scaffold numbers and positions were compiled for all SNPs with a PC loading value that exceeded 0.050. For each SNP of interest, 200 bp of flanking sequence was copied from the draft genome (Keeling et al., 2013). Cross‐referencing between the draft male and female genome assemblies was performed with BLAST+ (Camacho et al., 2008) to determine whether SNPs contributing to substructuring in the data were located on the same scaffolds in the male and female assemblies. SNPs of interest were checked against known protein sequence matches using BLAST2GO v4.0.2 on default settings (Conesa et al., 2005); gene ontologies for positive hits were investigated using UniProt.org (The UniProt Consortium, 2015; accessed Mar 10, 2018).

## 3 RESULTS

### 3.1 Alignment and filtering

A total of 30 low‐quality samples were removed, leaving 175 wild‐collected and 13 laboratory‐bred samples (*N* = 188) for further analysis. After trimming barcodes and adapters, we obtained 255 million reads of 62 bp in length from 188 samples. On average, 85% of reads were successfully mapped to the reference genome. Quality scores for Run 1 (HiSeq) and Run 2 (NextSeq) were similar, with average phred scores of 36 and 34, respectively. On average, Run 2 had 47% more unique read locations per sample than Run 1, but average read depths in Run 2 were 39% lower. The consistency and reproducibility of GBS across both genotyping platforms is supported by Campbell et al. (2017).

Using the draft female reference genome, STACKS yielded 18,503 SNPs for the unfiltered dataset (Table 1). After removal of loci with 5% MM and MAF (i.e., 5%‐only treatment), a total of 2,077 SNPs remained in the 5%‐only dataset. Further filtering for HWE removed 207 SNPs, and LD filtering removed an additional 388 SNPs from the female‐aligned dataset, leaving a total of 1,480 SNPs in the FF dataset. Results for the male reference genome were similar (Table 1).

### 3.2 Principal components analysis

**Figure 2:**
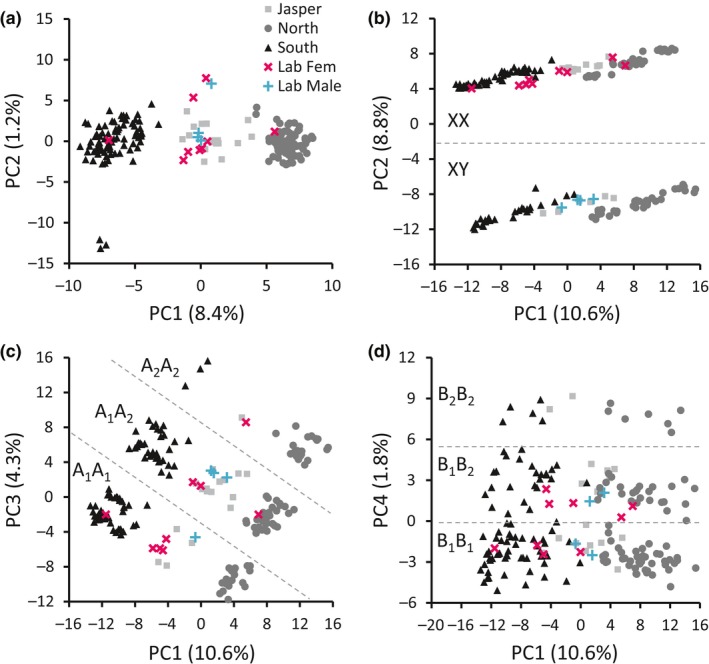
Principal component analyses of 175 wild‐caught and 13 laboratory‐bred MPB aligned to the female MPB genome. (a) FF dataset with 1,480 SNPs filtered at 5% MM, 5% MAF, HWE (*p* = 0.000025), LD (*r* ^2 ^= 0.5). (b–d) 5%‐only dataset with 2,077 SNPs filtered at 5% MM and 5% MAF, showing PC1 × PC2, PC1 × PC3, and PC1 × PC4, respectively

The FF treatment represents a widely accepted approach to filtering datasets for population genetics questions. The PCA of this set of SNPs showed clustering of individuals by geographic location (mainly latitude) of sampling sites, with a central cluster comprised of samples from Jasper National Park and the majority of laboratory‐bred north–south crosses (Figure 2a) (Trevoy et al., 2018). All PCA results were replicated using data aligned to the male MPB reference genome, where similarly partitioned patterns were found (Supporting Information Figure S1). A single female laboratory‐bred specimen was found in each of the distinct north and south clusters (Figure 2a). The PC2 axis did not appear to relate to geography, separating three of 12 samples collected in 2014 near the town of Canmore, Alberta, from the larger southern cluster.

**Figure 3:**
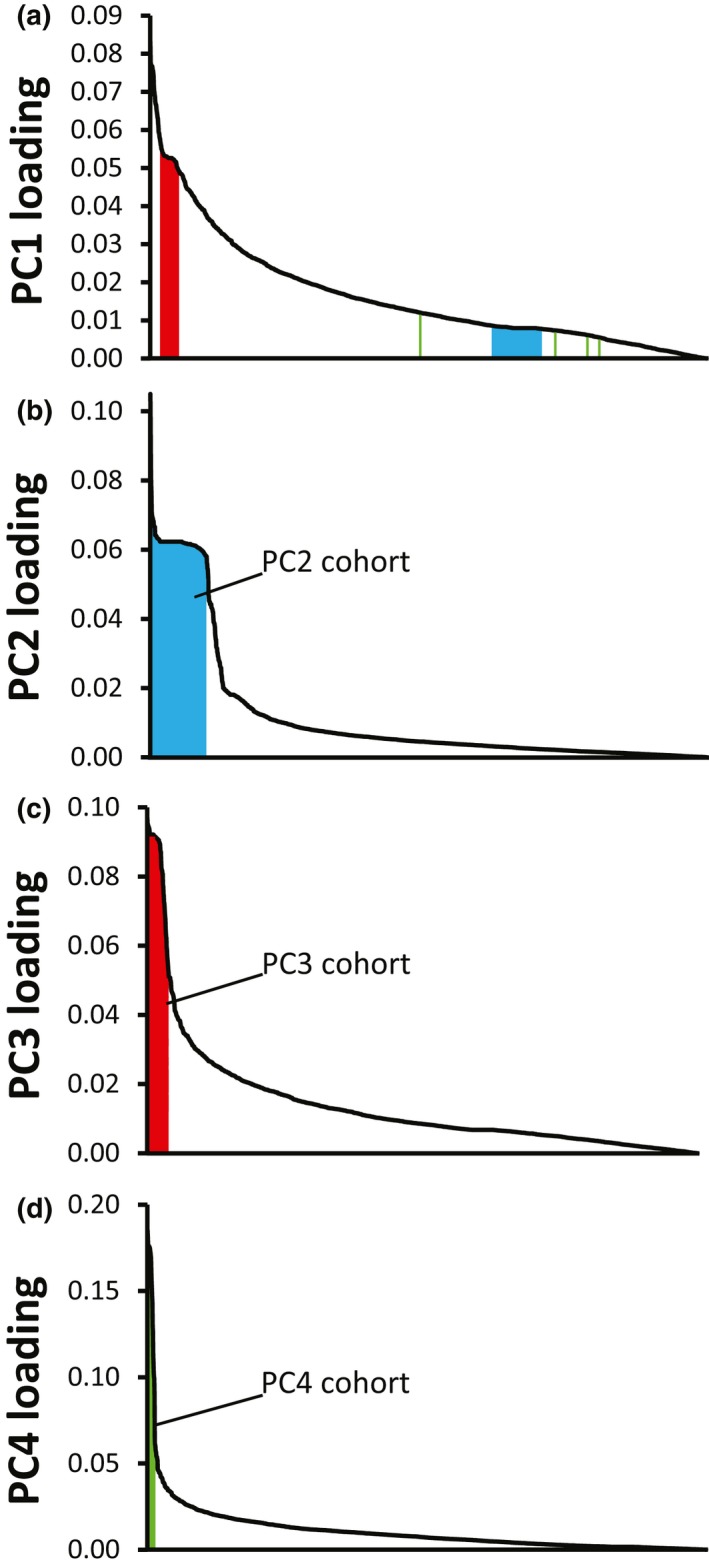
Principal component loadings arranged in descending order for Axes 1–4 of 175 wild‐caught MPB, 2,077 SNPs, 5% MM, 5% MAF. Locations within PC1 for loci contributing heavily to PCs 2, 3, and 4 are shown in blue, red, and green, respectively

In contrast, the 5%‐only dataset aligned to the female MPB genome showed the effect of including SNPs that violated the LD and HWE assumptions. In this PCA plot, the north–south division was reflected in the PC1 axis, but the PC2 axis showed strong nongeographic clustering (Figure 2b). PC2 clustered individuals into two groups, with 68 (39%; upper cluster) individuals clearly separated from another group of 107 (61%; lower cluster) (Figure 2b). While loadings on the PC1 axis showed a relatively smooth decline (Figure 3a), PC2 loadings contained a plateau of 217 loci with values exceeding 0.050 when viewed in descending order of PC loadings (Figure 3b). These 217 loci were located on 62 scaffolds on the draft female reference genome, with 56% of the SNPs concentrated on just 10 scaffolds (Table 2). This cohort of highly weighted loci showed a large difference in allele frequency between the two clusters of samples. The individuals in the upper cluster of Figure 2b were almost uniformly heterozygous at each of the 217 loci (99.3%), while those in the lower cluster were almost uniformly homozygous (99.9%). Of the thirteen laboratory‐bred individuals, all male beetles were found in the upper cluster while all females were in the lower one (Figure 2b). A separate dataset consisting of 157 laboratory‐bred, morphologically sexed MPB specimens contained an axis of similar size that sorted individuals by sex with 98% accuracy (data not shown). The cohort of loci with PC2 loadings of >0.050 accounted for 10.4% of all genotyped loci in the dataset that was filtered only at 5% MAF and 5% MM. These patterns were largely consistent even with varying MAF and MM. For example, 6%–12% of loci remained in this cohort when refiltering at various combinations of MAF (2%–20%) and MM (0%–50%), and when subsampling by subpopulation, genotyping batch, or collection year (data not presented).

**Table 2:** Scaffold distribution of SNPs that contribute significantly to a PC axis (>0.050 PC loading) from a PCA on the 5%‐only dataset aligned to the female MPB genome. Numbers indicate how many separate draft genome scaffolds contain SNPs contributing to that PC, with successive rows indicating more SNPs on each scaffold. Only SNPs that are exclusive to PC1 are included in that column; SNPs that are shared with PC3 are included only in the column for PC3

SNPs per Scaffold	PC1 Scaffolds	PC1 SNPs	PC2 Scaffolds	PC2 SNPs	PC3 Scaffolds	PC3 SNPs	PC4 Scaffolds	PC4 SNPs
1–2	33	39	39	48	14	16	5	5
3–5	3	9	13	47	2	8	1	3
6–9	0	0	5	37	1	8	0	0
10–14	0	0	2	24	0	0	1	10
≥15	0	0	3	61	2	56	1	19
Total	36	48	62	217	18	88	8	37

The PC3 axis for the 5%‐only dataset divided samples into groups that, when viewed in combination with the PC1 axis, gave nine clusters arranged diagonally (Figure 2c). Clustering was determined by 88 highly weighted loci (PC loading >0.050) (Figure 3) that were associated partially with north/south sampling location. MAF differed by 80% between the highest (A_2_A_2_) and lowest (A_1_A_1_) clusters (Figure 2c). Between northern and southern samples, MAF differed by 25%. These 88 loci were on 18 scaffolds in the draft female reference genome, with 64 (73%) of the loci concentrated on three unique scaffolds (Table 2). Additionally, 56 of this cohort of 88 SNPs were included within the highly weighted loci from the PC1 axis (Figure 3a). Similar to the PC3 axis, the PC4 distribution was influenced by 37 high‐weight SNPs, although the clustering of specimens in the PC1 × PC4 plot was less apparent (Figure 2d). Most of the loci (78%) comprising the high‐weight PC4 cohort were located on two unique scaffolds (Table 2). No high‐weight loci were shared between the PC2 cohort and those for PCs 1, 3, or 4 (Figure 5).

### 3.3 LDna results

**Figure 4:**
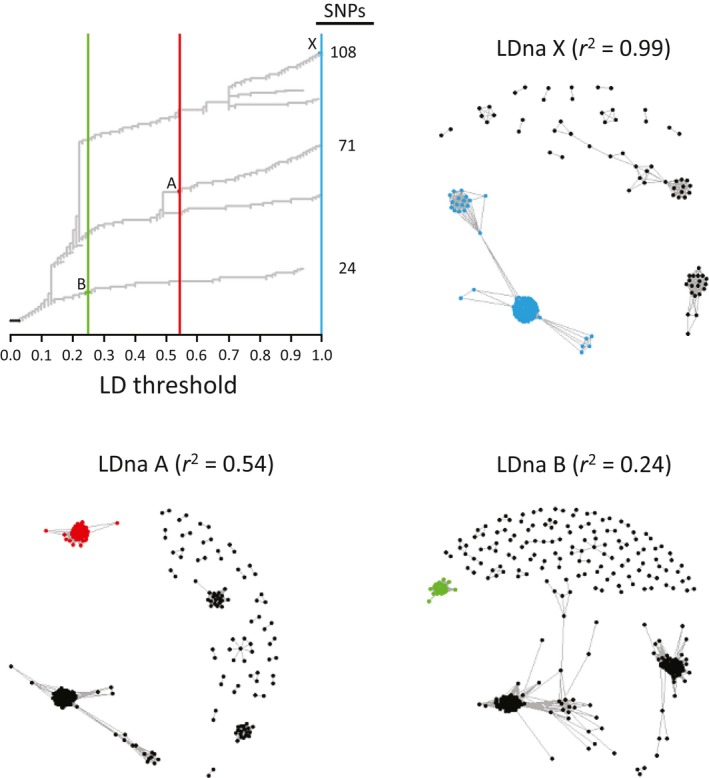
Linkage disequilibrium network analysis (LDna) for 2,077 SNPs, filtered at 5% MM and 5% MAF. Number of edges (e) is equal to 10, and cluster splitting (φ) is equal to 2. Clustering is depicted as a treespace progressing with increasing support for LD, as indicated by *r* ^2^. LDna cohort X at *r* ^2 ^= 0.99, LDna cohort A at *r* ^2 ^= 0.54, and LDna cohort B at *r* ^2 ^= 0.24 are highlighted in blue, red, and green, respectively, as they appear along the treespace

**Figure 5:**
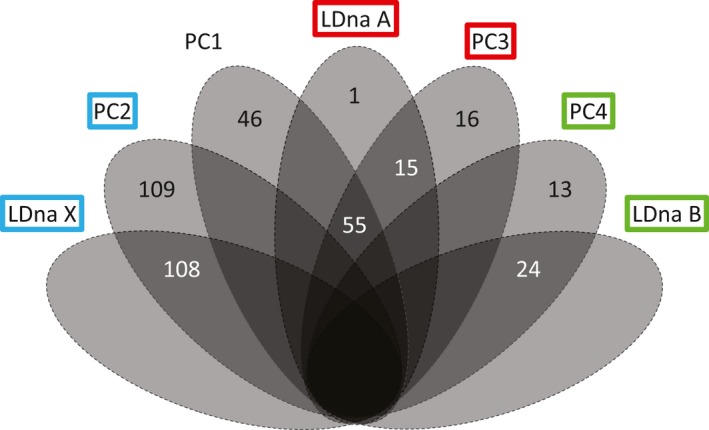
Correspondence among SNPs with high contributions to PCs 1, 2, 3, and 4 and LDna cohorts X, A, and B, based on analysis of 2,077 SNPs in 175 MPB samples from BC and Alberta. SNPs are treated as contributing to an axis if their PC loading weight exceeds 0.050. Combinations with 0 markers are left blank. Two SNPs, which were shared between PC1 and PC3 but not LDna cohort A, are not shown

Linkage disequilibrium network analysis was used to visualize mutually exclusive cohorts of putatively linked loci. Analysis of the 5%‐only (2,077 SNPs) dataset revealed six SNP cohorts (Supporting Information Figure S2). We focused on three of the six described cohorts that contained more than 21 loci (1% of the total data) (Figure 4). These three LD cohorts, designated LDna X (108 loci), LDna A (71 loci), and LDna B (24 loci), had 100%, 99%, and 100% of their SNPs also occurring in the PC2, PC3, and PC4 high‐weight SNP cohorts, respectively (Figure 5).

### 3.4 BLAST results

We identified a total of 390 SNPs with high PC loadings within the 5%‐only dataset. These SNPs were derived as: 48 SNPs from the PC1 axis only; 217 SNPs from PC2; 88 SNPs from PC 3; and 37 SNPs from PC4 (PC loadings >0.050). However, three SNPs were removed because the variant was too close to the edge of a reference scaffold to extract a flanking sequence of more than 50 bps. Thus, a total of 387 SNPs from the 5%‐only dataset were used for gene ontology analyses.

Using BLAST2GO, we found matching gene annotations for 140 unique proteins (Table 3). The annotations were related to molecular‐level activities performed by gene products for 51.4% and 46.3% of SNPs in the PC1 and PC3 cohorts, respectively. The largest portion of genes annotated for the PC2 cohort (44.8%) was components of larger biological processes accomplished by multiple molecular activities, such as oxidation and reduction. Annotations for the PC4 cohort were evenly split between molecular functional genes and biological processes, at 42.9% for each (Table 3). At least 12 of the 83 different proteins found for PC2 were related to neurotransmission, either as structural components of neurons or as essential components in the regulation and propagation of signals within the synaptic cleft (Supporting Information Table S1b). The gene annotations for the PC3 cohort included genes for microfilament binding, vesicle formation, and transport of vesicles along microfilaments (Supporting Information Table S1c). No single biological process was noticeably well represented for the PC1 and PC4 cohorts (Supporting Information Table S1a,d). The greatest number of annotated hits matched *T. castaneum *and *Anoplophora glabripennis *Motsch (Supporting Information Figure S3). Of the hits matching the *T. castaneum *genome, 79% from the PC2 cohort were located on chromosomes 2 and 4; 69% from PC3 were from chromosome 6; and 70% from PC4 were from chromosome 3 (Supporting Information Table S2).

**Table 3:** Gene ontologies for SNPs with significant contributions to PCs 1–4 (PC loading >0.050). PC1 refers only to loci that did not overlap with PC3. Percent given after/for cellular, molecular, and biological gene ontology categories include unique ontology results only. Cellular components refer to cellular structures in which a gene product performs a function, molecular functional refers to genes with molecular‐level activities performed by gene products, and biological processes refer to larger processes accomplished by multiple molecular activities

	PC1	PC2	PC3	PC4
Total loci	48	214	88	37
Annotated loci	18	93	40	13
Unique proteins	16	83	29	12
Unique Gene Ontology Terms	37	183	95	42
% cellular components	5/13.5	36/19.7	14/14.7	6/14.3
% molecular functional genes	19/51.4	65/35.5	44/46.3	18/42.9
% biological processes	13/35.1	82/44.8	37/38.9	18/42.9

## 4 DISCUSSION

### 4.1 Overview

In bioinformatics, the choice of filtering methods is informed by the needs of the experimental question (Schilling et al., 2014). The SNP dataset shown here was used previously to discern population structure in MPB (Trevoy et al., 2018), but continues to provide a basis for further genomics research. Here, we describe a method to uncover genomic regions of interest for future research of gene function and evolution. PCAs of our minimally filtered dataset revealed both nongeographic and geographic clustering of samples (Figure 2b,c) driven by mutually exclusive cohorts of SNP loci in tight LD (Figure 4). Comparison between LD network analysis and loadings from PCA showed three major cohorts of SNPs, including one large cohort associated with beetle sex, a second associated loosely with sampling location, and a third with no obvious biological associations (Figure 5).

### 4.2 Population genetic structure

When filtered for HWE and LD (i.e., FF dataset), PCA results support a north–south geographic division among the sampling locations (Figure 2a), in agreement with prior studies (e.g., Samarasekera et al., 2012; Janes et al., 2014; Batista et al., 2016; Trevoy et al., 2018). As demonstrated in Trevoy et al. (2018), the Jasper population is intermediate to the north and south populations. This suggests a geographic area of hybridization, either from converging invasive fronts meeting in Jasper, or as a result of an existing intermediate population from British Columbia forming a third front of eastward invasion. We find further support for the intermediate nature of Jasper in the placement of laboratory‐bred, north–south hybrid specimens, which are intermingled with the Jasper population. The female laboratory‐bred specimens in both the north and south clusters could be the result of pre‐emergence mating among siblings within a bolt, a known occurrence in MPB (Bleiker, Heron, Braithwaite, & Smith, 2013; Janes et al., 2016).

### 4.3 Nongeographic clustering—possible sex‐linked paralogues in MPB

Datasets that were not filtered based on LD (i.e., the 5%‐only) showed additional clustering that did not clearly correspond to sampling locality. The PC2 axis sharply segregated individuals by percent heterozygosity based on 217 SNP loci that had high loadings. The homozygous group contained all the female individuals from the sexed, laboratory‐bred specimens (Figures 2 and 4b) and included 61% of all samples, while morphologically sexed laboratory‐bred males grouped with the heterozygous PC2 cohort. The division among sexed individuals is consistent with the female‐biased sex ratio observed by other researchers in MPB (64%, McGhehey, 1969; 62%, Safranyik, 1976; 61%, Lachowsky & Reid, 2014). We hypothesize that the PC2 axis is driven by recent nucleotide substitutions in sex‐linked genes located on the neo‐XY chromosomes, with heterozygous loci indicating males, which are the heterogametic sex.

The neo‐X chromosome in MPB is thought to be a fusion of the largest ancestral autosome and the ancestral X chromosome, leaving the daughter autosomal chromatid to become the neo‐Y after the loss of the ancestral Yp chromosome (Lanier, 1981; Zúñiga et al., 2002). This fusion with sex chromatids either inhibits or suspends the autosomal portions from crossing over between sexes, transforming the formerly linked autosomal chromatids into evolutionarily and functionally distinct units (Kaiser & Bachtrog, 2010; Steinemann & Steinemann, 1998; Turner, 2005). Thus, point mutations and fixation of previously variable loci from the ancestrally autosomal fragments would have proceeded independently on each new fused chromosome (Kimura 1962; Rice, 1996). However, sections of the neo‐Y chromosome may still align with homologous regions of the neo‐X scaffolds, creating paralogous SNPs.

If the distinct groupings formed by the PC2 cohort are due to SNP paralogues on the historically autosomal portions of the neo‐XY complex, this may explain why homologous hits on the genome of *T. castaneum*, another beetle species, are located predominantly on autosomes. Of the 78 BLAST matches between the PC2 cohort and the *T. castaneum *genome*,* 80% were found on autosomes 2 and 4 (Supporting Information Table S2). Synteny between MPB and *T. castaneum* has been demonstrated (Keeling et al., 2013). However, the two species are widely separated by evolutionary history and karyogamy; evidence for shared autosomal ancestry is only suggestive at this point (Lanier & Wood, 1968; McKenna et al., 2015; Richards et al., 2008).

Despite support for neo‐XY paralogues as the source of sex‐associated SNPs, there is also evidence to the contrary. For example, scaffolds containing sex‐linked SNPs also include some SNPs that were not fully diagnostic for beetle sex. One explanation for this could be that these loci have not yet reached fixation in one or both MPB sexes. It is also possible that incomplete segregation is caused by one or more pseudoautosomal regions of the neo‐XY complex that may still undergo recombination (Charlesworth, Charlesworth, & Marais, 2005). More work is needed to determine if the sex chromosomes of *D. ponderosae* cross over during cell division, as in many other species of plants, animals, and fungi (Blavet et al., 2012; Otto et al., 2011). In any case, our imputed sex‐linked scaffolds do not include those predicted by Keeling et al. (2013), who suggested six different scaffolds based on their reduced SNP content per kbp. A linkage map or a complete genome sequence assembly for MPB would provide more definitive evaluation of these sex‐linked scaffolds.

The finding that PC2 is associated with sex has various implications and applications. If true, it can be expected that paralogues constitute 6%–12% of any given SNP dataset for MPB. Organisms with a neo‐XY mechanism like MPB, therefore, pose a unique case for filtering. These paralogous data violate the assumption of locus independence that is commonly applied in population genetics analyses, and these loci may be removed with LD filtering. However, these same evaluations of LD can also provide valuable insight into genomic architecture.

Despite the challenges inherent in filtering paralogous data, these putative neo‐XY markers would be useful for determining the sex of samples. Due to the narrow temporal window for collecting postemergence adults, most field samples of MPB are collected in the late larval stage (Carlson & Cole, 1965; Safranyik, 1968; Safranyik & Carroll, 2006), which shows no obvious sexual dimorphism. Within our own analysis, beetles were not sexed prior to genotyping due to the high proportion of larval individuals. Traditional MPB sexing methods (i.e., stridulation and seventh tergite morphology; Lyon, 1958) are time‐consuming and have some degree of inaccuracy (Rosenberger et al., 2016). Both methods call for undamaged adult beetles, but stridulation, a behavioral indicator, further requires specimens to be alive. Meanwhile, genetic methods can be employed on various life‐history stages and on physically damaged specimens (Stovall et al., 2018). While there is a genetic means of sexing MPB using microsatellites (Davis et al., 2009), our results demonstrate a SNP‐based sexing method that is easily applied to NGS datasets without the additional cost and labor required to genotype microsatellites. Reliable sexing of MPB is valuable for monitoring and predictive modeling of MPB outbreaks because sex ratio skew is related to outbreak maturity (James, Janes, Roe, & Cooke, 2016).

### 4.4 PC 3—candidate for adaptive selection?

Unlike the PC2 cohort of SNPs, the SNPs detected by PC3 do not cluster individuals by imputed gender; rather the PC3 axis has substantial geographic signal (Figure 2c). The PC3 axis is instead driven by variation in a subset of SNPs already found to contribute significantly to PC1 (Figure 3). LD network analysis shows that LDna SNP cohort A is 96% identical to the portion of the high‐weight PC3 cohort that overlaps with high‐weight PC1 SNPs (Figure 5). This axis is therefore unrelated to sex, but may form an island of genomic differentiation within the geographic signal of the PC1 axis that is concentrated on five autosomal scaffolds of the female MPB genome (Table 2). This result complements recent work on divergence in the neo‐Y chromosome as a mechanism for speciation (Bracewell et al., 2017; Dowle et al., 2017). Adding to these studies, our high‐weight SNP cohorts from PC axes 1, 3, and 4 provide evidence of autosomal divergence across the Canadian range of MPB.

BLAST2GO analysis suggests that a disproportionate number of the genes associated with the geographically informative PC3 cohort may relate to biological processes of intracellular transport and transcription, but are not linked by ontology or pathway (Supporting Information Table S1c). A possible explanation is that there has been concatenation of adaptive genes into a higher‐impact QTL, or supergene—a group of different genes, although often related, that are closely packed on the genome and inherited together. Supergenes were first described for flower morphology in plants (Hermann et al., 2013; Mather, 1950; Yeaman & Whitlock, 2011), but are also key determinants in the coloring of several insect species (Brown & Benson, 1974; Clarke, Sheppard, & Thornton, 1968; Joron et al., 2011; Lindtke et al., 2017; Nijhout, 2003). More conclusive evidence of a multi‐gene QTL could make MPB one of the first species described with a metabolic, rather than structural, supergene.

While the differences between northern and southern demes could provide evidence of unique selection pressure, a genomic inversion within one of the populations might also explain why spatially linked loci might appear to be under selection (Giglio et al., 2001; McCutcheon & von Dohlen 2011). An inversion of genomic sequence does not preclude the existence of selection pressure or a supergene, but does provide an alternative, neutral mechanism. Linkage disequilibrium may also arise through random genetic drift without any functionally active selection (Ohta, 1982). Further study of the genes implicated in the detected linkage cohorts could help explain the beetles’ expansion into northern Canada through mechanisms like adaptation in metabolic pathways. However, a full linkage map or genome assembly is necessary to determine if the differences between populations are indeed spatially related and whether they are a result of chromosomal inversion.

### 4.5 Integrating PCA with LD network analysis

Linkage disequilibrium network analysis detected at least three sizable cohorts of associated markers (Figure 4), each of which corresponds with an axis of the PCA on SNPs that were only lightly filtered for missing data and minor allele frequency (5%‐only dataset) (Figure 5). Standard filtering for LD removed these axes. There was substantial concentration of SNPs on a few draft genome scaffolds involved with PC cohorts 2, 3, and 4 and with LDna cohorts X, A, and B, respectively (Table 2). Further exploration of genomic differentiation in MPB, using integrated PCA and LD analysis, may discriminate additional SNP cohorts (Supporting Information Figure S2; Table S3).

It may be possible to apply this method to other SNP datasets to detect correlated genomic differentiation in subsets of SNPs by (a) partitioning genetic variance among individuals in a PCA and examining the distribution of PC loadings, and (b) discrimination of SNP cohorts with LD network analysis to verify that correlated SNP cohorts are due to linkage disequilibrium, rather than population structuring. However, studies using more conventional approaches to detect divergence between populations are required to verify the efficacy of this method (Lindtke & Yeaman, 2017). Studies using simulated data, with different taxa, traits, sample sizes, and loci are also necessary to evaluate the robustness and generality of our method. We note that for MPB, the PCA step found more SNPs in each cohort than analysis by LDna alone, while LDna found almost no SNPs that were not in the PC cohorts.

While useful as a means of ensuring independence of loci in classical population genetics surveys, LD analysis can also offer insights into genomic architecture and differentiation, even within non‐model species (Baird, 2015; Barton, 2011; Kemppainen et al., 2015). Recent work by Li et al. (2018) has explored the potential to augment genome‐wide association studies (GWAS) in model organisms by imputing loci of interest using PCA to reduce complexity in large datasets, followed by linkage network analysis. Here, we demonstrate an independently developed version of such a method as a tool to detect genomic islands of differentiation in wild populations. The combination of PCA and LDna to detect cohorts of correlated SNP variation has allowed us to circumvent the need for precise knowledge of genomic positions. The use of a draft genome for our research, although useful in supporting our results, was not a requirement for the larger component of our analysis; similar analyses to those shown here are possible with a de novo dataset. Although the approach described here is less precise than a genome scan (see Turner et al., 2005; Renaut et al., 2013; Feulner *et al.* 2015), it offers a means to explore divergence in populations without the need for detailed knowledge of genomic locations, and with the benefit of preexisting or lower‐cost genetic marker datasets.

## 5 CONCLUSION

Our geographic survey of GBS SNP variation in the mountain pine beetle in western Canada has allowed us to determine both population structure and genomic architecture, as well as to explore functional aspects of population divergence. In addition to replicating previously documented population structure, we uncovered at least three cohorts of genomically linked loci when we dispensed with the traditional approach to filtering for HWE and LD.

The largest cohort of linked SNPs is hypothesized to be composed of paralogous loci from the neo‐X and neo‐Y regions of the sex chromosomes. This provides a means to determine the sex of individuals. The second SNP cohort is composed of geographically associated loci in tight LD. This SNP cohort yielded several candidate genes for further study of adaptive radiation and selective pressures facing MPB as it expands eastward in Canada. A third cohort of SNPs is independent of the other two and represents further opportunities for research. Using a procedure related to that of Li et al. (2018) to integrate principal components analysis and linkage disequilibrium analyses, we describe a novel approach that can potentially be applied to the burgeoning number of reduced representation SNP datasets to find putative islands of genomic differentiation in non‐model species.

## CONFLICT OF INTEREST

None declared.

## AUTHOR CONTRIBUTIONS

The experiments were conceived and designed by F.A.H.S. and J.K.J. The laboratory work was performed by S.A.L.T. and J.K.J. The data were analyzed by S.A.L.T and K.M., including analytical tools developed by K.M. The data were interpreted by S.A.L.T. and F.A.H.S. The paper was written by S.A.L.T., F.A.H.S., and J.K.J.

## Data Availability

In‐house PERL wrapper scripts available at https://github.com/muirheadk/GBS_analysis_pipeline. Snapshot and SNP data are available on Dryad under https://doi.org/10.5061/dryad.jn8hj0t.
