# Supplementary material for: Repurposing population genetics data to discern genomic architecture: A case study of linkage cohort detection in mountain pine beetle (Dendroctonus ponderosae)
Source: Ecol Evol. 2018 Dec 26;9(3):1147–59. doi: 10.1002/ece3.4803 (PMC6374669; doi:10.1002/ece3.4803)
Supplement: Supplementary file 1 [file ECE3-9-1147-s001.pdf]

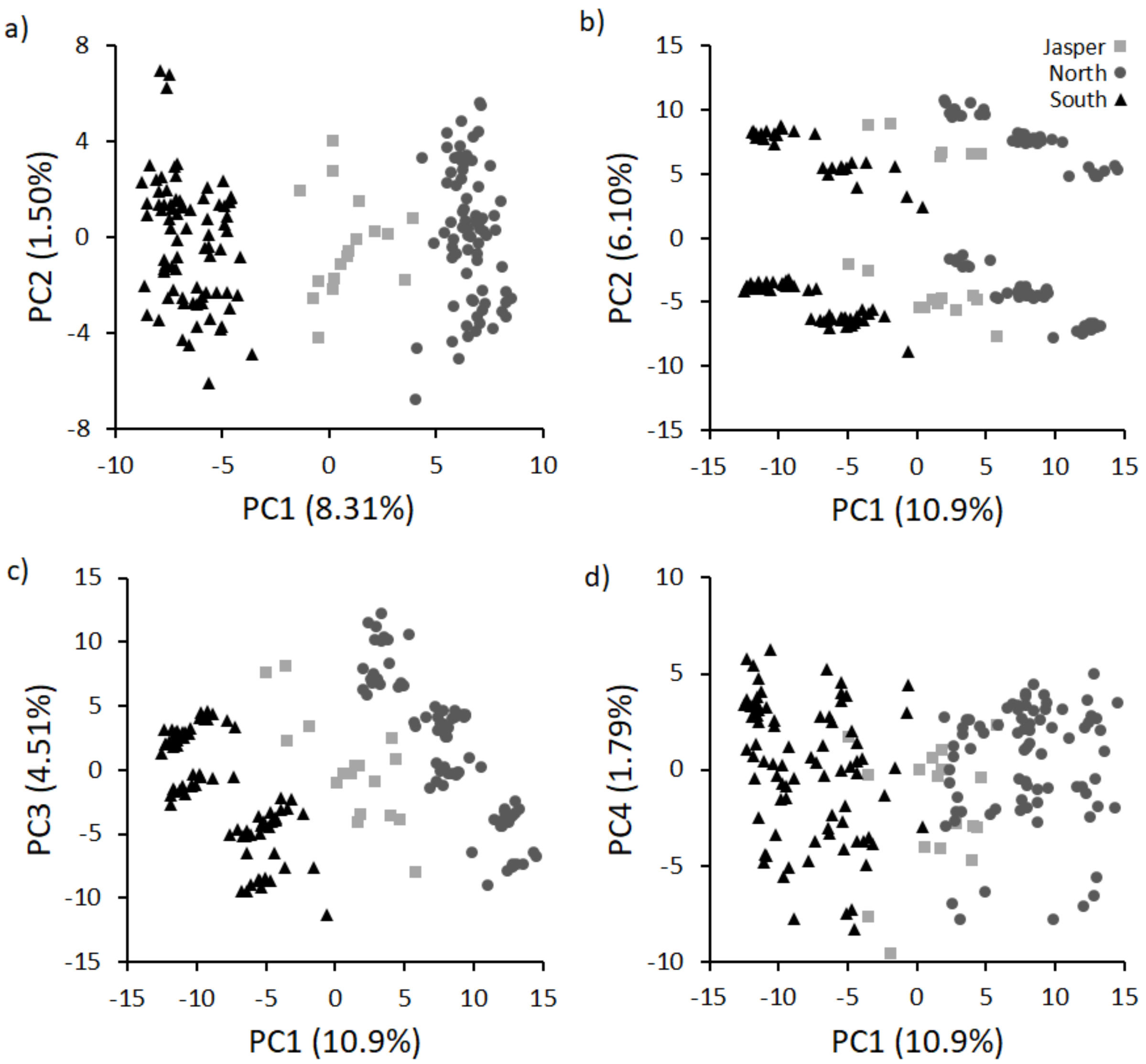

Supplementary Figure 1. Principle component analyses of 175 wild-caught MPB aligned to the male MPB genome. a) FF dataset with 1488 SNPs filtered at 5% MM, 5% MAF, HWE (p=0.000025), LD (r^2=0.5). b-d) 5%-only dataset with 1908 SNPs filtered at 5% MM and 5% MAF, showing PC1 x PC2, PC1 x PC3, and PC1 x PC4, respectively.

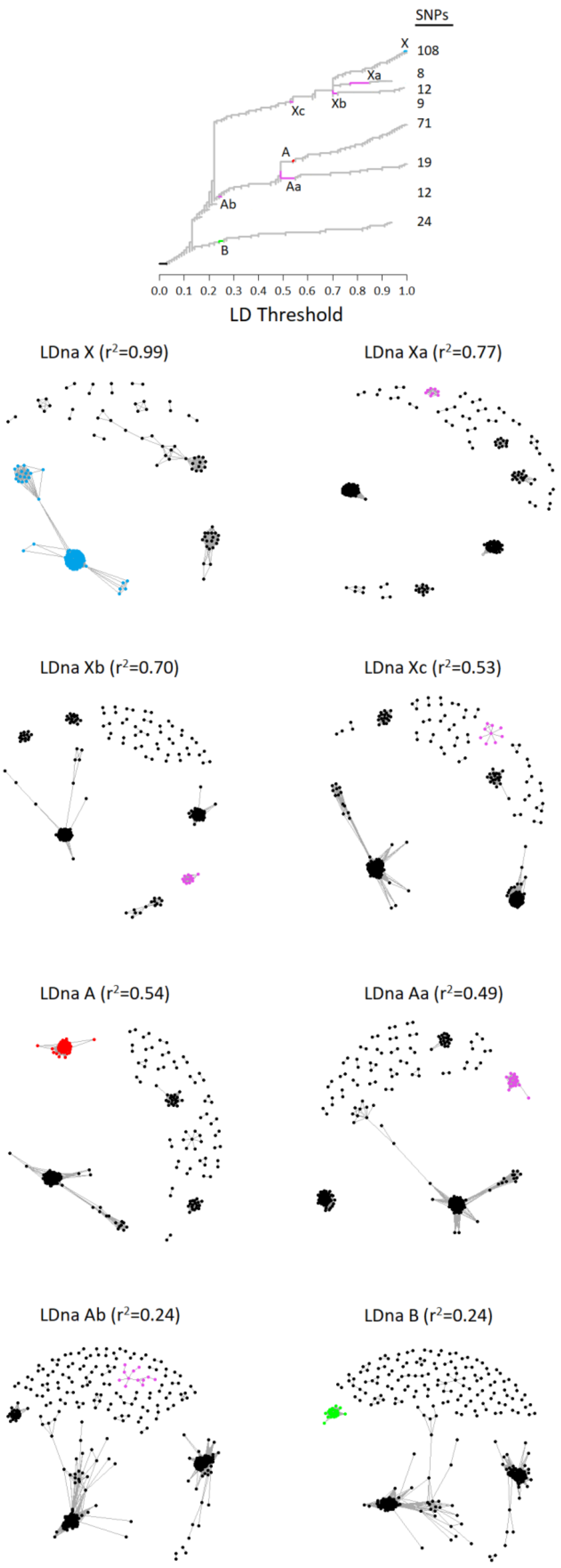

Supplementary Figure 2: Expanded linkage disequilibrium network analysis (LDna) for 2077 SNPs, 5% MM, 5% MAF, aligned to the female draft genome. Number of edges (E) is equal to 10, Cluster splitting (φ) is equal to 2. Clustering is depicted as a treespace progressing with increasing support for LD, as indicated by r2. Cohort X at r^2 = 1.00, Cohort A at r^2 = 0.54, and Cohort B at r^2 = 0.24 are highlighted in blue, red and green, respectively, as in Figure 3. All other highlighted cohorts are comprised of fewer than 20 SNPs and are highlighted in purple.

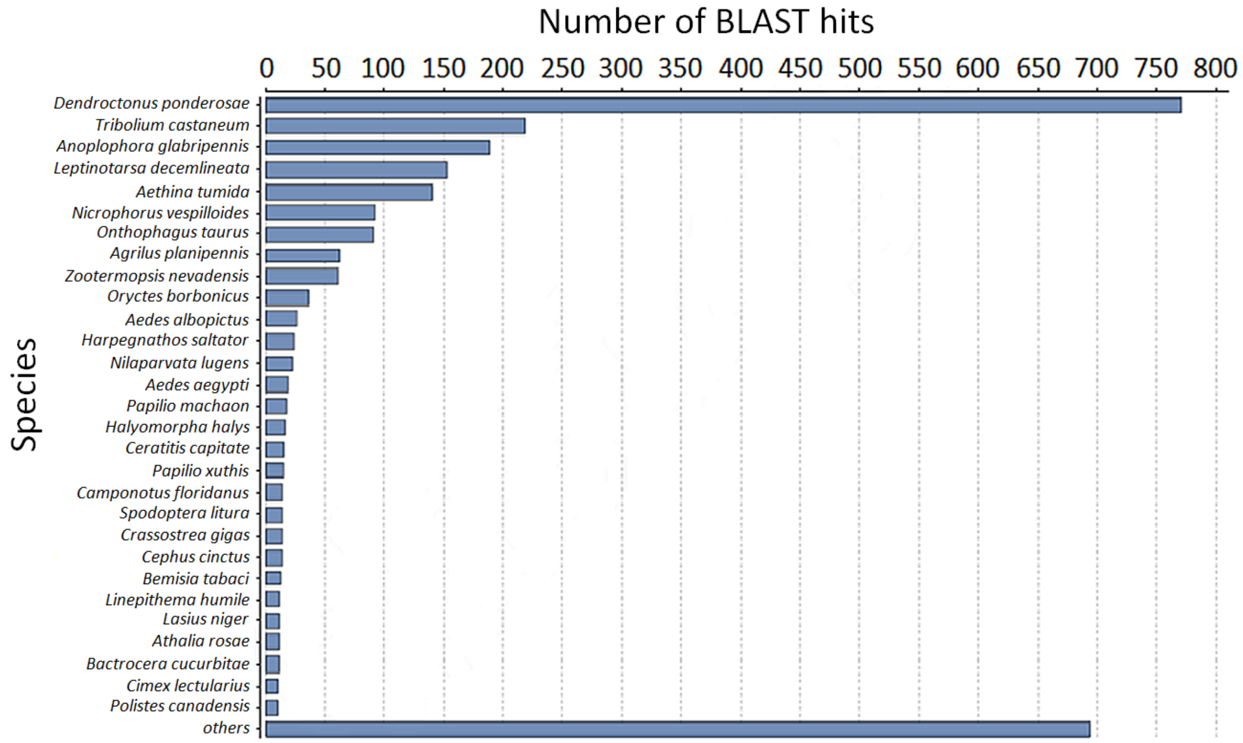

Supplementary Figure 3. Distribution of species identified in a BLASTn search of 303 sequences of 200bp from loci with high-weight loadings on the PC2 and PC3 axes of a PCA for *Dendroctonus ponderosae*.
